# Advances in oral disease models: a mini-review of developments from 2015 to 2025

**DOI:** 10.3389/fdmed.2026.1864810

**Published:** 2026-06-08

**Authors:** Jia Huang, Justin Le-Tran, Nika S. Kobayashi, Yoshifumi Kobayashi, Emi Shimizu

**Affiliations:** 1 School of Graduate Studies, Newark Health Science Campus, Rutgers, Newark, NJ, United States; 2 Department of Oral Biology, Rutgers School of Dental Medicine, Rutgers, Newark, NJ, United States

**Keywords:** artificial intelligence, epithelial-immune crosstalk, multispecies biofilms, oral disease models, organ-on-chip, patient-derived organoids, precision medicine, translational research

## Abstract

Oral diseases represent one of the most widespread global health burdens, affecting billions of people worldwide, causing pain, disability, and substantial treatment costs. Despite their prevalence, progress in prevention and therapy has been limited, in part, by experimental models that do not fully capture the complexity of the oral biological and environmental landscape. Over the past decade, however, major advances in model development have expanded the possibilities for studying oral disease. This mini-review summarizes advances from 2015 to 2025, focusing on caries and endodontic infections, gingivitis and periodontitis, peri-implantitis, mucosal disorders, oral and oropharyngeal cancers, and salivary gland diseases. Recent innovations include saliva-derived biofilm systems that reproduce ecological transitions, organ-on-chip systems that replicate fluid dynamics, and patient-derived organoids and xenografts that preserve clinical characteristics. In parallel, immune-integrated models now allow direct interrogation of host responses to pathogens. Separate from these experimental platforms, advanced analytical and computational approaches, including single-cell profiling, spatial transcriptomics, radiomics, and artificial intelligence (AI)-assisted image analysis, are increasingly linking molecular signatures with structural and functional disease outcomes. Together, these experimental models and complementary analytical tools mark a shift from reductionist approaches toward dynamic, patient-relevant frameworks that better capture the complexity of oral diseases. Remaining challenges include modeling chronic disease progression, incorporating viral and autoimmune components, and improving reproducibility through standardization across platforms. Addressing these limitations will be important for translating next-generation experimental models into clinically meaningful advances in oral health care.

## 1 Introduction

Oral diseases affect more than 3.5 billion people worldwide, making them among the most common yet often overlooked global health problems (1). Conditions such as dental caries, periodontal diseases, and oral mucosal disorders carry a heavy cost, both financially and in terms of quality of life. Oral and oropharyngeal cancers are especially serious, with survival rates dropping below 50% in advanced stages (2). Yet progress in prevention and treatment has been constrained by the limitations of traditional research models. Conventional bacterial cultures, two-dimensional cell lines, and simplified animal studies often fail to capture the full complexity of the oral cavity. This environment is shaped by diverse microbial communities, three-dimensional tissue architecture, immune interactions, and continuous mechanical forces (3–5).

To address these gaps, oral disease modeling has substantially advanced over the past decade. Organ-on-chip systems now allow researchers to track barrier function under physiologically relevant flow conditions (6–9). Patient-derived organoids and patient-derived xenografts (PDXs) retain individual tumor characteristics, offering promise for personalized medicine (10–12). Multispecies biofilms better reproduce ecological changes over time (13, 14), and immune-integrated models enable direct interrogation of host-pathogen interactions (15–17). Complementing these experimental models, analytical and computational approaches, such as single-cell sequencing, spatial transcriptomics, and artificial intelligence (AI)-assisted image analysis, are increasingly linking molecular details to whole-tissue outcomes (18–21).

**Figure 1:**
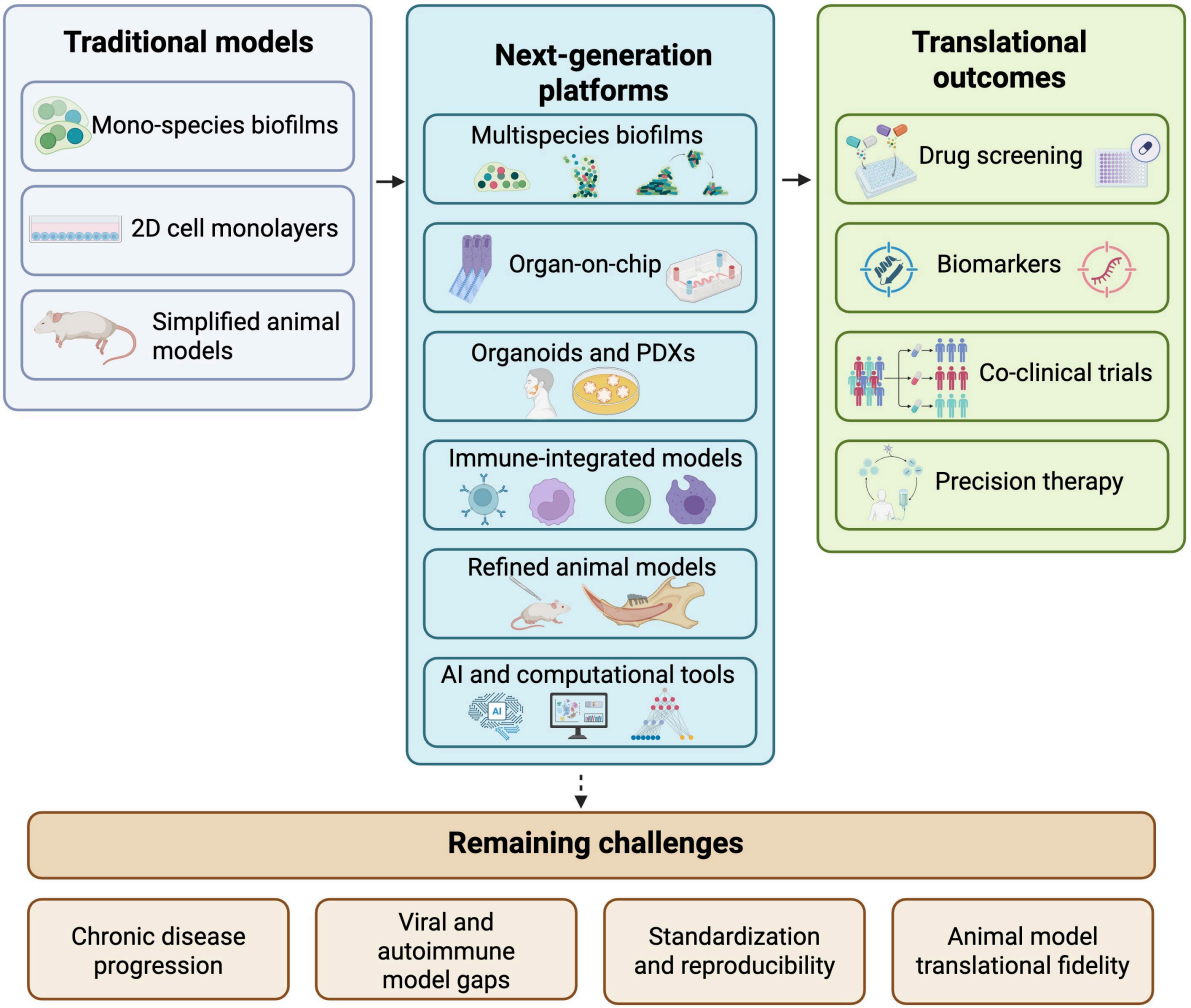
Evolution of oral disease modeling platforms toward clinical translation. Schematic overview of the shift from traditional models, including mono-species biofilms, two-dimensional cell monolayers, and simplified animal models, toward next-generation experimental platforms such as multispecies biofilms, organ-on-chip systems, patient-derived organoids and xenografts, immune-integrated models, and refined animal models. Artificial intelligence (AI)-assisted tools are shown as complementary analytical approaches that support image analysis, data integration, drug-response prediction, biomarker discovery, co-clinical trials, and precision therapy. These advances support translational applications including drug screening, biomarker discovery, co-clinical trials, and precision therapy. Key remaining challenges include chronic disease modeling, viral and autoimmune disease gaps, standardization, reproducibility, and animal model translational fidelity. Created in BioRender. Huang, J. (2026) https://BioRender.com/tb6jkjw

This mini-review provides an overview of progress from 2015 to 2025 across six major areas: dental caries and endodontic infections, gingivitis and periodontitis, peri-implantitis, mucosal diseases, oral cancers, and salivary gland disorders. For each area, we describe key advances, highlight shared challenges, and summarize potential future directions for the next generation of disease models aimed at improving the prevention, diagnosis, and treatment of oral diseases (Figure 1 and Supplementary Table S1). This mini-review emphasizes representative and translationally relevant platforms rather than exhaustive coverage of all available systems.

### 2.1 Biofilm and caries models

Caries models have evolved from single-species cultures toward complex, ecologically realistic systems spanning *in vitro*, *in situ*, and *in vivo* platforms (8, 22–24). Saliva- or plaque-derived microcosm biofilms cultivated in artificial mouth systems have become widely used to model caries-related microbial ecology (8, 22, 23, 25, 26). These systems can reproduce sucrose-driven dysbiosis (25, 27, 28), pH gradients (29, 30), and reproducible lesion endpoints (25, 27, 29, 30). Recent methodological advances include cross-kingdom incorporation of Candida albicans and salivary pellicle conditioning (27, 30), as well as continuous-flow configurations that better simulate oral hydrodynamics (29–31).

*In situ* intraoral appliance models allow biofilm development under genuine salivary and dietary conditions, making them particularly valuable for validating lesion progression and preventive agent efficacy, though inter-subject variability limits their scope (32–37). Animal models, especially rodent and gnotobiotic systems, remain the primary *in vivo* platforms for studying causal relationships among microbial communities, diet, and caries formation. They also provide immune and tissue-level context that no *in vitro* system can fully replicate (38–43). *Ex vivo* hemi-mandible preparations offer a pragmatic middle ground, preserving native tissue architecture without requiring live animals (39, 40).

Despite this progress, important limitations persist across all caries model systems. *In vitro* oral biofilm models remain limited in their ability to recapitulate the dynamic and complex *in vivo* environment (8, 22, 28, 44, 45), particularly with respect to real-time environmental monitoring (23, 25, 30) and integration of host components (27, 46). Animal models differ from humans in salivary composition and enamel microstructure, complicating direct translational extrapolation. Standardization remains a field-wide challenge. Unlike endodontic research, which benefits from consensus frameworks such as the 15-species Zurich model (47), caries studies still lack shared protocols for inoculum preparation, substrate conditioning, and flow conditions (44). Integrating microfluidics, biosensing, and computational modeling may ultimately enable more predictive and standardized caries research across all levels of biological complexity. Standardization is a recurring theme across model systems and is discussed at a field-wide level in Section 3. Taken together, caries models have translational value for studying caries-related dysbiosis and testing preventive strategies, but their clinical applicability remains mainly preclinical or validation-oriented because host integration, inter-subject variability, and protocol standardization remain unresolved.

### 2.2 Endodontic disease models

Building on advances in caries and biofilm research, endodontic disease models have progressed in parallel, with many approaches adapted from or inspired by developments in plaque ecology and multispecies biofilm systems. Organ-on-chip devices now integrate native dentin barriers with microfluidics to study real-time cellular and molecular responses at the dentin-pulp interface (6, 48–51). Some platforms track growth factor diffusion, such as transforming growth factor-*β* (TGF-*β*), or include neurovascular co-cultures to more closely mimic pulp tissue architecture and function.

Biofilm modeling within endodontic research has also advanced substantially. Standardized multispecies consortia, such as the 15-species Zurich biofilm model, are now widely used, showing how interspecies interactions and dentin tubule colonization influence antimicrobial resistance (47, 52, 53). These models highlight the limitations of earlier single-species systems, as mature biofilms formed on dentin exhibit markedly reduced susceptibility to sodium hypochlorite. Complementary imaging tools including optical coherence tomography (53), confocal laser scanning microscopy (53–55), and mechanical testing (56) link biofilm structure (55–57), dentin interactions (55, 56), and treatment efficacy (52, 54, 57–59).

*Ex vivo* systems retain anatomical complexity while allowing controlled experimental manipulation. Transparent root canal models, combined with computational fluid dynamics, help explain how irrigants behave in complex canal geometries (55–60). Whole-tooth organ cultures can now maintain pulp vitality for extended periods, supporting studies of reparative dentinogenesis and dentin bridge formation (61–64).

*In vivo*, rodent pulpitis models distinguish reversible from irreversible inflammation through macrophage polarization, offering predictive value for vital pulp therapy outcomes (65–67). Larger-animal studies of regenerative endodontic protocols have demonstrated the formation of vascularized, innervated pulp-like tissue capable of functional restoration (68–72).

Despite this progress, current systems often lack immune integration (73, 74), precise control of hypoxia and pH within biofilm models (75), and consistent translation from ectopic to orthotopic regenerative outcomes (5, 76, 77). Addressing these gaps will be critical for developing models that not only replicate disease processes but also guide effective regenerative therapies. Additional challenges include the lack of experimental validation for computational fluid dynamics simulations of irrigant behavior (78), and unresolved trade-offs between ectopic rodent implants and orthotopic or large-animal models for regenerative studies (76, 77). The lack of consensus on what constitutes “functional” regeneration is a cross-cutting issue discussed in Section 3. For endodontic disease, current models are valuable for testing irrigants, biomaterials, and regenerative strategies at the dentin-pulp interface, but clinical applicability is strongest for *ex vivo* disinfection and material testing, while organ-on-chip and regenerative platforms remain mainly preclinical because immune integration, microenvironmental control, and functional regeneration endpoints remain incomplete.

### 2.3 Gingivitis and periodontitis models

Research on gingivitis and periodontitis models has evolved from short-term, reductionist systems to more dynamic platforms that better reflect human disease development. Early reconstructed human gingiva models demonstrated that tissues respond differently to commensal vs. pathogenic biofilms (17, 79–81). Newer iterations now sustain host-microbe co-cultures for up to one week, highlighting adaptive epithelial barrier strengthening and induction of antimicrobial responses (82). Building on this foundation, perfused bioreactor “periodontal pocket” systems have incorporated fluid flow, fibroblasts, and immune cells, producing complex secretome profiles that more closely resemble inflamed periodontal tissues (83, 84).

Organ-on-chip technology has further advanced model sophistication. Gingival-crevice-on-chip devices recreate physiologic fluid flow and demonstrate flow-dependent microbial clearance (85), while vascularized gingival barrier chips maintain long-term stability with tunable inflammatory states (86, 87). Gum-on-chip platforms are beginning to incorporate immune cell recruitment and support anaerobic periodontal pathogens, a long-standing challenge in maintaining dysbiotic pathogenic communities (88). In parallel, humanized scaffolds-based systems that mimic tooth-gum architecture also improve ecological fidelity by replicating oxygen gradients characteristic of the periodontal niche (82, 88, 89).

Animal models remain important, especially with refinements in ligature techniques (90–93) and high-resolution micro-computed tomography readouts (90, 91, 93–95). Improved microbial colonization strategies now generate chronic infections that more closely resemble clinical periodontitis and link dysbiosis-driven Th17 immune expansion to alveolar bone loss (96).

Human experimental gingivitis studies provide valuable benchmarks, mapping early mediator shifts, interindividual variability in host responses, and potential systemic effects of localized inflammation. Together, these *in vitro*, *ex vivo*, *in vivo*, and human models (97, 98) form a continuum that increasingly reflects ecological complexity (99–104) and immune-microbial interactions (105–111), strengthening their relevance for mechanistic insight (97–99, 106, 110) and translational research (100, 109, 112).

Standardization challenges affecting the periodontal field are discussed alongside other subfields in Section 3. In periodontal research, these models help link biofilm dysbiosis with epithelial, immune, and bone-loss outcomes, but clinical applicability is currently strongest for human experimental gingivitis and animal bone-loss benchmarking, while personalized therapeutic prediction remains limited by chronicity and host variability.

### 2.4 Peri-Implantitis models

Peri-implantitis research has advanced along multiple complementary lines. Ligature-based models remain the backbone of the field because they predictably induce peri-implant bone loss (113–117); however, newer rat and mouse systems with standardized implants and harmonized protocols have improved reproducibility and cross-study comparability. These refinements have also shown that the timing of implant placement can shape disease severity, with immediate placement often accelerating peri-implant inflammation and tissue breakdown (115, 118–121).

At the same time, infection-driven models have become increasingly prominent. In murine systems, oral exposure to *Porphyromonas gingivalis* revealed that implants are more susceptible to dysbiotic challenge than natural teeth, with repeated infections promoting both microbial imbalance and peri-implant bone resorption (122). Similar approaches in rats, using polymicrobial gavage or direct inoculation with human-derived microbial consortia, have created disease phenotypes that include systemic immune features, more closely mirroring human peri-implantitis (123–125).

For capturing clinical signs such as probing depth, suppuration, and circumferential bone defects, large animal models, especially in dogs and minipigs, remain essential (126–128). The incorporation of systemic comorbidities, including diabetes, has underscored how systemic host health can influence peri-implant disease progression and has provided testbeds for targeted therapies (123, 127, 129, 130).

Meanwhile, *in vitro* biofilm systems grown on titanium surfaces have offered a controlled way to track the shift from health to peri-implant disease (131). Collectively, these complementary approaches bring the field closer to recapitulating the complexity of human peri-implantitis. From a translational perspective, peri-implantitis models are useful for reproducing peri-implant bone loss and testing material-, antimicrobial-, and host-risk interventions, but clinical applicability remains limited by species differences, protocol heterogeneity, and incomplete replication of human microbiota and comorbidities.

### 2.5 Models of Non-neoplastic oral mucosal diseases

Experimental models for non-cancerous oral mucosal diseases have become increasingly refined, particularly in the study of wound healing and microbial infection. Full-thickness three-dimensional (3D) oral mucosa equivalents, built from stratified keratinocytes layered over fibroblast-populated collagen hydrogels, now reliably reproduce native tissue architecture, basement membrane composition, and site-specific differentiation. These systems have been widely used in wound-repair studies, where re-epithelialization follows timelines that closely match human hard-palate wounds, with predictable cytokine and growth factor dynamics (132–136). At the clinical interface, a standardized intraoral wound challenge model (137) has become a key translational benchmark. It links preclinical models to human biology by enabling assessment of wound closure, tissue gene expression, salivary proteomics, and microbiome shifts (133, 138–140).

Organ-on-chip systems further enhance physiological relevance by introducing fluid flow, enabling real-time monitoring of barrier function under conditions that more closely approximate the oral mucosal environment (46, 141). In parallel, mechanobiology studies have shown that extracellular matrix stiffness and composition directly influence epithelial cohesion, migration, and wound closure. These findings highlight the role of biophysical cues in maintaining mucosal homeostasis and guiding tissue repair (15, 142).

Immune-integrated modeling has also advanced substantially. Incorporating macrophages into oral mucosa equivalents produces robust, pharmacologically responsive inflammatory signals and supports detailed immune profiling (15). Co-culture systems combining peripheral blood mononuclear cells with biofilms further highlight how epithelial cells mediate immune responses, underscoring their importance for physiological accuracy (143).

Despite these gains, important gaps persist in models of viral oral diseases, including herpes simplex virus infection and non-oncogenic human papillomavirus (HPV)-associated conditions. Autoimmune blistering diseases may need dedicated organotypic systems capable of replicating features such as acantholysis or subepithelial splitting (144, 145). Further incorporation of immune components, together with *ex vivo* and *in vivo* large-animal systems, could support the continued development of this field. A more detailed discussion of viral and autoimmune model gaps appears in Section 3.

Several advanced readouts could improve mucosal disease models, including optical coherence tomography, spatial transcriptomics, and real-time biosensing for pH or oxygen. Wider use of these tools would provide deeper mechanistic insight and strengthen translational relevance. For non-neoplastic oral mucosal diseases, these models are valuable for wound-healing, barrier-function, and host-microbe studies, but most organotypic and organ-on-chip systems remain preclinical because vascular, neural, immune, viral, and autoimmune components are still incompletely modeled.

### 2.6 Oral and oropharyngeal cancer models

Over the past decade, models of oral cavity and oropharyngeal squamous cell carcinomas (OC/OP SCCs) have become increasingly refined. These advances reflect the need for site-specific approaches that account for differences in anatomy, etiology, and clinical behavior (146–150). A wide range of experimental models now supports this effort, including patient-derived organoids, PDXs, *ex vivo* tumor slices, immunocompetent animal models, and engineered 3D microenvironments. In parallel, computational approaches and AI-assisted tools are increasingly being used to support tumor classification, treatment-response prediction, and cross-platform data integration.

Organoids have evolved from proof-of-concept systems to clinically relevant platforms. They preserve genetic, transcriptomic, and histologic fidelity (11, 151–155), support medium-throughput testing of chemotherapeutics and radiotherapy (152, 155–158), and increasingly correlate with patient outcomes. Oropharyngeal SCC-specific organoid libraries have demonstrated that organoid formation rates and chemoradiotherapy resistance patterns align with clinical response, underscoring their predictive value (153). Large-scale efforts have improved oral squamous cell carcinoma (OSCC) organoid success rates (152, 159), incorporated cancer-associated fibroblasts, and created multicenter repositories that stratify by tumor protein p53 (TP53) and HPV16 status (151). These resources now link radio response patterns with clinical behavior, strengthening the case for organoids as prospective clinical decision-support tools (157, 160–162).

*Ex vivo* tumor slice cultures deliver same-week drug and radiation assessments while preserving tumor architecture and immune elements (163–165). PDXs of OP SCC retain histologic and genomic traits for radiosensitivity and biomarker studies (166–168), though HPV-positive PDXs remain difficult to establish due to Epstein–Barr virus (EBV)-driven lymphoma contamination (169–172).

Immunocompetent models have filled critical gaps: carcinogen-induced tumors in mice demonstrate that radiation efficacy depends on intact immunity (173–178), and engineered HPV16-driven orthotopic models in humanized mice now support immunotherapy and vaccination studies (179, 180).

3D systems, including spheroids, organotypic co-cultures with cancer-associated fibroblasts, and collagen-based scaffolds that recreate hypoxia gradients, more accurately model tumor-microenvironment interactions and drug responses than monolayer cultures (181). Microfluidic platforms integrating tumor, stromal, and immune components offer further potential for personalized testing (159, 182–185).

Among computational approaches, radiomics has shown strong potential for predicting HPV and p16 status across multicenter cohorts (186). AI-assisted tools are also being used to classify organoid identity (187) and integrate functional-response and genomic data to guide more precise, patient-specific therapeutic strategies in OC/OP SCC (187–189). In this context, AI-assisted approaches serve less as disease models themselves than as analytical tools that extract quantitative features from model-derived images, radiologic datasets, organoid morphology, and multi-omics profiles (18–21, 184, 185). These applications may improve patient stratification, functional drug-response prediction, and comparison between experimental platforms, particularly when paired with patient-derived organoids and integrated genomic or functional-response datasets (149, 151–153, 156, 157, 159, 160). However, most AI-assisted workflows remain exploratory because they depend on relatively small or institution-specific datasets, often lack external validation, and are not yet standardized for routine preclinical or clinical decision-making.

As organoids advance toward prospective clinical validation, quality-control gaps persist. A methodological review of published head and neck squamous cell carcinoma (HNSCC) organoid studies reveals that short tandem repeat (STR) authentication, mycoplasma screening, and HPV verification beyond p16 immunohistochemistry are inconsistently reported. Several leading biobank studies determine HPV status primarily through p16 staining or anatomical site inference rather than confirmatory molecular testing (190, 191), and explicit reporting of STR profiling and mycoplasma certification remains variable. These observations align with broader concerns highlighted by international standardization efforts, which note the absence of universally agreed quality-control standards for organoid production (192). Key model-selection debates remain unresolved, particularly for HPV-positive oropharyngeal cancer, where trade-offs between engraftment efficiency, immune fidelity, and throughput complicate platform choice for immunotherapy testing (190, 193). Among the categories reviewed, OC/OP SCC models are among the closest to clinical translation, supporting drug and radiation testing, biomarker discovery, and patient stratification; however, organoids, *ex vivo* slices, and PDXs remain investigational because establishment rates, quality control, immune fidelity, and external validation remain incomplete.

### 2.7 Salivary gland disease models

Recent advances in salivary gland disease models increasingly integrate animal and human systems with expanded assessment of functional outcomes. Models of radiation-induced xerostomia have progressed from simple single-dose approaches to clinically relevant fractionated irradiation protocols that mirror clinical cancer treatment. These approaches now include quantitative saliva flow monitoring, fibrosis assessment, and immune profiling (194–197). Salivary cytokines have emerged as predictors of late toxicity (194), and precision-guided murine platforms (198, 199) allow standardized testing of regenerative therapies. Mechanistic studies have highlighted the critical roles of SRY-box transcription factor 2 (SOX2)-positive progenitors and parasympathetic innervation in acinar regeneration (200), leading to biomaterial strategies such as oxidized-alginate hydrogels that deliver muscarinic agonists to restore glandular function (201, 202).

Human-derived platforms have progressed in parallel. Patient-derived salivary gland organoids now demonstrate acinar function through calcium flux and swelling assays, responding to cholinergic and cystic fibrosis transmembrane conductance regulator (CFTR) agonists (203, 204). Notably, organoids generated from patients with Sjögren disease show therapeutic responsiveness to phosphodiesterase 4 (PDE4) inhibitors, directly linking disease tissue with therapeutic drug testing (205). Collagen-based organoids have achieved transplantation success and functional recovery in irradiated murine models (206), while Good Manufacturing Practice (GMP)-optimized processes are accelerating clinical translation (207). In large-animal studies, minipig models enable transplantation of human progenitors that integrate with vasculature and nerves under immunosuppression (208).

Autoimmune disease modeling has broadened beyond the non-obese diabetic (NOD) mouse system to include epithelial-driven systems (209–212). Viral-vector mediated lysosome-associated membrane protein 3 (LAMP3) overexpression produces progressive hyposalivation with autoantibodies (209), and acinar-specific tumor necrosis factor-α (TNF-α) expression drives chronic inflammation and functional decline (211). Humanized immune system models (212) and inducible ectopic lymphoid structures (210) further enhance disease relevance by capturing key immunopathologic features.

Evaluation criteria now cover structural integrity, functional performance, secretory and ion transport, immunity, and regenerative capacity, supported by single-cell and spatial transcriptomic analyses (201, 203, 209, 213). Altogether, these advances have built a versatile toolkit that connects mechanistic insight with translational and clinical applications in salivary gland research.

Overall, salivary gland disease models are valuable for evaluating secretory dysfunction, radiation injury, autoimmune mechanisms, and regeneration, but clinical applicability remains emerging because functional recovery benchmarks, immune-neural integration, and clinical validation of organoid or transplantation outputs remain incomplete. The challenge of defining “functional” recovery beyond histological repair is discussed in Section 3.

## 3 Future Perspectives for the Development of Oral Disease Models

Several cross-cutting priorities will shape the next generation of oral disease models. First, standardization and reproducibility remain major challenges across platforms. Caries biofilm studies still lack shared protocols for inoculum preparation, substrate conditioning, and flow conditions, while endodontic research has benefited from reproducible frameworks such as the 15-species Zurich biofilm model (44, 47). Similar consensus is emerging for patient-derived organoid platforms, where quality-control guidelines are being developed for authentication, mycoplasma testing, and viral status verification (192). Extending comparable standards to gingival, peri-implant, mucosal, and regenerative models will be essential for improving cross-study comparability.

Second, regenerative fields need clearer definitions of “functional” recovery. In endodontic (5, 76), salivary gland (201, 203, 209), and periodontal tissue models (87), histological repair alone may not reflect true restoration of vascularization, innervation, secretory function, immune competence, or tissue-specific performance. Multidimensional benchmarks and longitudinal monitoring will be needed to distinguish tissue regeneration from structural mimicry.

Third, better models are needed for viral and autoimmune oral diseases. Stratified epithelial systems may improve herpes simplex virus type 1 modeling, and emerging *in vitro* platforms are beginning to address non-oncogenic HPV (214–217). Autoimmune mucosal diseases, including pemphigus vulgaris, mucous membrane pemphigoid, and Behçet's disease, require models that reproduce acantholysis, basement membrane disruption, complement activation, and immune-mediated injury (133, 144, 146).

Fourth, future models should better capture chronic disease progression and temporal dynamics (26, 218). Current systems often emphasize early infection or short-term responses (80, 82), whereas oral diseases frequently involve gradual dysbiosis (219, 220), hypoxia, nutrient limitation (52, 221), chronic inflammation, treatment response, and tissue remodeling (222, 223).

Finally, tiered translational frameworks that integrate experimental models, including animal models, organ-on-chip systems, and patient-derived organoids, with computational approaches may improve clinical relevance (224, 225). In this framework, humanized mouse models (226, 227), gnotobiotic systems (228), and advanced *in vivo* imaging (229) can capture systemic and host-microbe interactions, whereas machine learning (230), radiomics (186), and fluid dynamics modeling (231, 232) can support drug-response prediction, patient stratification, and biomimetic model design. These computational approaches may also help integrate imaging data, spatial omics, organoid-response data, and readouts from microphysiological systems across platforms. However, their translational value will depend on larger annotated datasets, external validation, transparent reporting, and standardized performance metrics. Together, these approaches can help oral disease models become more reproducible, functionally informative, and clinically meaningful.
